# Comparison of Open Albumin Dialysis (OPAL) With Prometheus Fractionated Plasma Separation and Adsorption (FPSA) and Standard Medical Treatment for Acute‐On‐Chronic Liver Failure

**DOI:** 10.1111/aor.14977

**Published:** 2025-02-25

**Authors:** Justa Friebus‐Kardash, Amina Louzi, Andreas Kribben, Hartmut H. Schmidt, Michael Jahn, Bartosz Tyczynski, Jassin Rashidi‐Alavijeh, Andreas Schütte, Amos Zeller

**Affiliations:** ^1^ Department of Nephrology University of Duisburg‐Essen, University Hospital Essen Essen Germany; ^2^ Department of Gastroenterology, Hepatology and Transplant Medicine University of Duisburg‐Essen, University Hospital Essen Essen Germany

**Keywords:** acute‐on‐chronic liver failure (ACLF), bilirubin, CLIF‐C‐ACLF score, MELD score, open albumin dialysis (OPAL), Prometheus

## Abstract

**Background:**

Acute‐on‐chronic liver failure (ACLF) is associated with high short‐term mortality of up to 40%. Albumin dialysis is a therapeutic option that can be used to bridge patients with ACLF to liver transplantation or recovery.

**Methods:**

This retrospective cohort study was conducted to determine the effectiveness and adverse effects of open albumin dialysis (OPAL) by comparing the biochemical and clinical variables of model for end‐stage liver disease (MELD)‐matched ACLF patients who received one of three treatments: OPAL plus standard medical treatment (SMT; 22 patients), Prometheus dialysis fractionated plasma separation and adsorption (FPSA) plus SMT (41 patients), or hemodialysis plus SMT (24 patients) at the University Hospital Essen.

**Results:**

OPAL treatment significantly reduced liver function tests such as bilirubin (*p* = 0.0001) and creatinine levels (*p* = 0.049). Therefore, OPAL therapy significantly reduced the MELD score (*p* = 0.001) and the Chronic Liver Failure Consortium (CLIF‐C) ACLF (*p* = 0.0005) score. In both extracorporeal liver support groups, the decrease in MELD score was significantly stronger than that achieved with SMT (OPAL vs. SMT, *p* = 0.002; Prometheus vs. SMT, *p* = 0.0001; OPAL vs. Prometheus *p* = 0.90). In comparison to the SMT group, survival rates after 14 and 30 days were significantly higher in the Prometheus group (*p* = 0.0008 and 0.03) and tended to be better in the OPAL group, although statistical significance was not reached (*p* = 0.06 and *p* = 0.11).

**Conclusions:**

Our analysis revealed OPAL is an efficient method of albumin dialysis yielding a reduction of bilirubin and creatinine levels and improving clinical scoring in ACLF patients. OPAL as well as Prometheus were associated with a stronger reduction of relevant biochemical variables of liver function and amelioration in clinical scoring in comparison to SMT. However, it should be considered that patients from the SMT group were older and experienced progressive ACLF with high mortality risks compared to the patients from the OPAL and Prometheus groups. Thus, when interpreting the study results, several limitations including small sample size and heterogeneity of the treatment groups due to the lack of randomization should be taken into account.

## Graphical abstract

The retrospective cohort study is the first to compare OPAL with Prometheus and hemodialysis for adult ACLF patients. Both extracorporeal liver support systems were comparably effective with regard to the reduction of bilirubin and creatinine levels surpassing the effects seen in hemodialysis. We recognized improvements in clinical scoring, such as the CLIF C‐ACLF score, due to OPAL and Prometheus treatment, and Prometheus significantly reduced short‐term mortality after 14 and 30 days compared to standard medical treatment and hemodialysis.

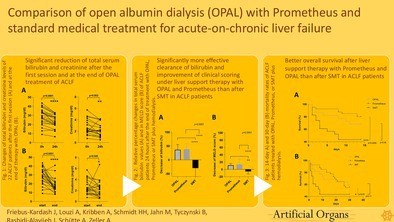

## 1 Introduction

Acute‐on‐chronic liver failure (ACLF) is a life‐threatening condition characterized by acute deterioration of preexisting chronic liver disease that consequently leads to the rapid development of extrahepatic organ failure and is accompanied by high short‐term mortality rates [1]. The European Association for the Study of the Liver (EASL) and the Chronic Liver Failure Consortium (CLIF‐C) provide their own definition of ACLF; this definition considers multiorgan failure to be a hallmark of ACLF according to the CLIF‐C organ failure score (CLIF‐C OF) [2, 3].

Currently, standard medical care is restricted to the treatment of precipitating factors, as well as the treatment of organ failures [1, 4]. Liver transplant is a life‐saving curative option [1, 3]; however, several extracorporeal liver support systems provide the possibility of restoring impaired liver function and bridging ACLF patients to liver transplant or liver recovery [5, 6]. The Molecular Adsorbent Recirculating System (MARS) and fractioned plasma separation and adsorption (FPSA; Prometheus dialysis) are two well‐studied liver support methods for ACLF patients [6]. These two devices effectively remove the water‐soluble and albumin‐bound toxins that accumulate in ACLF patients as a result of compromised liver function, but they are based on two different technical principles [6, 7]. The MARS system uses an additional albumin circuit prior to high‐flux hemodialysis to eliminate both water‐soluble and albumin‐bound toxins. The open albumin dialysis (OPAL) system is a further development of MARS that is more efficient than MARS in albumin‐bound detoxification due to a surface‐increased charcoal absorber enabling a higher concentration gradient between the blood and albumin dialysate [8, 9].

In contrast, the function of the Prometheus device combines plasma separation, adsorption, and traditional high‐flux hemodialysis [10]. The patient's blood passes through a 250‐kDalton filter that separates the plasma and extracts the albumin fraction that will be further detoxified in the secondary circuit by a neutral resin adsorber and an anion‐exchange adsorber [6, 10]. In 2021, the Prometheus system was withdrawn from the market in Germany and is no longer available. However, Prometheus is still used as a liver support device in other countries, especially in Asia [11].

The current retrospective analysis was conducted to evaluate the effectiveness of a new mode of albumin dialysis (OPAL) in clearing the albumin‐bound and water‐soluble toxins, improving clinical prognosis scores, and reducing short‐term mortality rates among ACLF patients in comparison to the Prometheus system and conventional hemodialysis.

## 2 Materials and Methods

### 2.1 Study Population

We performed a retrospective analysis of single‐center data from patients with the diagnosis of ACLF who were treated with one of two extracorporeal liver support systems or conventional hemodialysis. The OPAL group consisted of 22 ACLF patients who were treated between December 2021 and October 2023. The Prometheus group involved 41 ACLF patients who were treated between December 2012 and January 2021. The SMT group contained 24 ACLF patients who were treated between June 2013 and November 2022 with intermittent hemodialysis for hepatorenal syndrome. None of the included patients was admitted to an ICU during treatment sessions. Hepatorenal syndrome was diagnosed according to the established criteria of the International Ascites Club [12]. Patients treated consistently with the same extracorporeal liver support (ECLS) system were included in the analysis. According to our local standard operating procedures, OPAL and Prometheus were preferentially considered for patients with total bilirubin values higher than 10 mg/dL. Because the Prometheus dialysis device was not available at our center after the middle of 2021, OPAL was the treatment of choice thereafter. Patients without hyperbilirubinemia but with acute kidney injury related to hepatorenal syndrome underwent hemodialysis.

Clinical and biochemical covariates were manually collected by retrospective review of electronic medical records. We determined the MELD score at baseline and after the end of treatment for all patients retrospectively using the same pre‐2016 MELD score calculator based on the MELD formula from the model for end‐stage liver disease described by Kamath et al. [13]. All patients provided written informed consent before the initiation of any medical treatment. Ethical approval for this study was obtained from the local Ethics Board (23‐11 405‐BO).

### 2.2 Treatment of ACLF

OPAL therapy was performed almost daily. The OPAL device was attached to a standard hemodialysis machine, the FMC MultiFiltrate (Fresenius Medical Care AG, Bad Homburg, Germany), with a Maxicycler absorber (Albutec, Rostock, Germany). The albumin circuit was primed with 500 mL of a 20% human albumin solution. The OPAL device was built according to the manufacturer's instructions. The average treatment time was 360 min. Blood flow rates were set at between 100 and 150 mL/min, and albumin flow rates were set at 250 mL/min.

Prometheus dialysis combines fractionated plasma separation and adsorption (FPSA) with hemodialysis. The Prometheus device (Fresenius Medical Care AG, Bad Homburg, Germany) consists of two parts: a “standard” extracorporeal blood circuit connected to a double‐lumen dialysis catheter and a secondary circuit containing fractionated plasma. Both circuits are integrated into a modified hemodialysis unit (4008 H, Fresenius Medical Care AG, Bad Homburg, Germany) and are separately monitored by various hardware and software methods. A high‐flux hemodialyzer (F60S, Fresenius Medical Care AG, Bad Homburg, Germany) is used to remove water‐soluble substances. The patient's blood (flow rate, 200 mL/min) enters the first extracorporeal circuit and passes through an albumin‐permeable filter (Albu‐Flow, Fresenius Medical Care AG, Bad Homburg, Germany). The filtered albumin‐rich plasma then flows through a secondary circuit (volume approximately 450 mL) at a flow rate of 30 mL/min. The albumin‐rich plasma is purified from lipophilic albumin‐bound toxins by flowing through two adsorbers: direct adsorption of the albumin‐bound toxins takes place in a neutral resin adsorber (Prometh 01), whereas negatively charged toxins such as bilirubin are separated in an anion exchanger (Prometh 02) that uses chloride as the counterion. The average treatment time is 360 min.

Vascular access was obtained with a double‐lumen hemodialysis catheter placed in the jugular or femoral vein. Anticoagulation was maintained by the application of regional citrate or by systemic infusion of unfractionated heparin.

### 2.3 Statistical Analysis

Categorical variables were expressed as numbers and percentages. Continuous variables were presented in corresponding figures and tables as medians with interquartile ranges. We compared absolute numbers of categorical variables between two groups with a two‐tailed *χ* ^*2*^ test. Assuming an abnormal distribution, differences in continuous variables between the three liver support procedure groups were assessed with the Kruskal–Wallis test. Dunn's test was used as a post hoc test for multiple comparisons. For comparison of pretreatment and posttreatment values, the Wilcoxon test was applied. Kaplan–Meier point estimates of the survival probabilities were calculated on study days 14 and 30. Differences were considered statistically significant at the level of *p* < 0.05. Statistical analyses were performed with GraphPad Prism version 6 (GraphPad Software Inc., La Jolla, CA, USA) and IBM SPSS Statistics version 23 (IBM Corp., Armonk, NY, USA).

## 3 Results

### 3.1 Characteristics of Patients in the OPAL Group

In all, 22 patients with ACLF were treated with the OPAL device between December 2021 and October 2023. The patients' median age was 56 years (33–68 years), and most were men (73%) (Table 1). The main reasons for the development of ACLF in this group were continuous alcohol consumption (41%) and infections (32%) (Table 1). Most of the patients were found to have Grade 2 ACLF (73%); 18%, Grade 1; and 9%, Grade 3 ACLF (Table 1). Patients underwent a median of seven sessions of OPAL, with a median treatment duration of 6 h (Table 2). Before the initiation of therapy, the patients' median MELD score was 34, and their median CLIF‐C ACLF score was 49 points (Table 2). Tables 1 and 2 illustrate the patient characteristics of the OPAL cohort.

**TABLE 1:** Baseline characteristics of 22 ACLF patients treated with OPAL, 41 ACLF patients treated with Prometheus for liver support, and 24 ACLF patients treated with SMT plus hemodialysis.

Variable	OPAL *n* = 22	SMT *n* = 24	RR (CI)	*p* value	OPAL *n* = 22	Prometheus *n* = 41	RR (CI)	*p* value
Women, *n* (%)	5 (23)	11 (46)	0.5 (0.2–1.1)	0.1	5 (23)	11 (27)	0.85 (0.3–2.0)	0.72
Multiorgan failure, *n* (%)	19 (86)	24 (100)	0.86 (0.7–1.0)	*0.06*	19 (86)	30 (73)	1.18 (0.‐1.5)	0.23
Hepatorenal syndrome, *n* (%)	14 (64)	24 (100)	0.64 (0.4–0.8)	**0.001**	14 (64)	26 (63)	1.0 (0.7–1.5)	0.98
ACLF Grade 1, *n* (%)	4 (18)	12 (50)	0.37 (0.1–0.9)	**0.02**	4 (18)	11 (27)	0.68 (0.3–1.7)	0.44
ACLF Grade 2, *n* (%)	16 (73)	11 (46)	1.59 (1.0–2.7)	*0.06*	16 (73)	26 (63)	1.15 (0.8–1.6)	0.46
ACLF Grade 3, *n* (%)	2 (9)	1 (4)	2.18 (0.3–16.1)	0.50	2 (9)	4 (10)	0.93 (0.2–4.0)	0.93
ACLF due to infection, *n* (%)	7 (32)	16 (67)	0.48 (0.2–0.9)	**0.02**	7 (32)	21 (51)	0.62 (0.3–1.2)	0.14
ACLF due to bleeding, *n* (%)	1 (5)	3 (13)	0.36 (0.1–2.3)	0.34	1 (5)	4 (10)	0.47 (0.1–2.8)	0.47
ACLF due to alcohol consumption, *n* (%)	9 (41)	1 (4)	9.8 (1.8–57.8)	**0.003**	9 (41)	12 (29)	1.4 (0.7–2.7)	0.35
ACLF due to other reasons, *n* (%)	5 (23)	4 (17)	1.36 (0.4–4.2)	0.61	5 (23)	4 (10)	2.33 (0.7–7.3)	0.16
Hepatic encephalopathy, *n* (%)	9 (41)	12 (50)	0.82 (0.4–1.5)	0.54	9 (41)	17 (42)	0.99 (0.5–1.8)	0.97
Dialysis before therapy initiation, *n* (%)	5 (23)	6 (25)	0.9 (0.3–2.5)	0.86	5 (23)	11 (27)	0.85 (0.3–2.0)	0.72
Admission to ICU before therapy initiation, *n* (%)	10 (46)	12 (50)	0.91 (0.5–1.7)	0.76	10 (46)	23 (56)	0.81 (0.5–1.3)	0.42
Another liver support device before therapy initiation, *n* (%)	2 (9)	0 (0)	Infinity (0.6‐infinity)	0.13	2 (9)	1 (2)	3.73 (0.5–27.8)	0.24

Abbreviations: ACLF, Acute‐on‐chronic liver failure; CI, confidence interval; ICU, intensive care unit; OPAL, open albumin dialysis; RR, relative risk; RRT, renal replacement therapy; SMT, standard medical treatment.

**TABLE 2:** Comparison of the continuous clinical and laboratory values of 22 ACLF patients treated with OPAL, 41 treated with Prometheus for liver support, and 24 with SMT plus hemodialysis. All values are given as medians with interquartile ranges.

	OPAL *n* = 17	Prometheus *n* = 41	SMT *n* = 24	*p* value
Age, years	56 (41–61)	46 (40–61)	60 (50–69)	**0.01**
OPAL vs. Prometheus				ns
OPAL vs. SMT				ns
Prometheus vs. SMT				**0.01**
MELD score before therapy	34 (37–31)	33 (29–37)	32 (25–37)	0.43
CLIF‐C ACLF score before therapy	49 (47–55)	47 (41–51)	50 (47–57)	**0.02**
OPAL vs. Prometheus				ns
OPAL vs. SMT				ns
Prometheus vs. SMT				**0.04**
Number of therapy sessions	7 (3–9)	7 (5–11)	8 (3–11)	0.74
Blood flow (mL/min)	150 (100–150)	200 (200–200)	180 (150–250)	**0.0001**
OPAL vs. Prometheus				**0.0001**
OPAL vs. SMT				**0.0001**
Prometheus vs. SMT				ns
Bilirubin (mg/dL)	29.2 (22.3–34.8)	29 (23.4–33.8)	7.2 (2.0–20.2)	**0.0001**
OPAL vs. Prometheus				ns
OPAL vs. SMT				**0.0001**
Prometheus vs. SMT				**0.0001**
ALT (U/L)	62 (32–124)	57 (38–104	42 (24–165)	0.58
AST (U/L)	169 (109–234)	109 (66–162)	77 (26–197)	**0.005**
OPAL vs. Prometheus				**0.04**
OPAL vs. SMT				**0.005**
Prometheus vs. SMT				ns
AP (U/L)	185 (144–250)	173 (118–277)	127 (100–215)	0.11
GGT (U/L)	225 (104–465)	117 (69–237)	123 (59–203)	0.06
LDH (U/L)	272 (200–423)	245 (199–315)	283 (212–467)	0.3
Creatinine (mg/dL)	2.1 (1.7–3.6)	2.1 (1.3–3.6)	5 (3.4–6.3)	**0.0001**
OPAL vs. Prometheus				ns
OPAL vs. SMT				**0.003**
Prometheus vs. SMT				**0.0002**
Urea‐N (mg/dL)	47.6 (27.7–86.5)	42.9 (27.0–69.8)	93 (60.4–142.5)	**0.002**
OPAL vs. Prometheus				ns
OPAL vs. SMT				**0.01**
Prometheus vs. SMT				**0.003**
Albumin (mg/dL)	3.2 (2.8–3.7)	3.2 (3.0–3.3)	3.1 (2.5–3.3)	0.41
Hemoglobin (g/dL)	9.1 (8.2–27.7)	8.9 (7.7–10.4)	8.4 (7.5–10.4)	0.57
Thrombocytes (/nL)	113 (66–256)	103 (72–153)	97 (52–141)	0.47
Prothrombin time (%)	42 (30–52)	43 (35–53)	44 (33–56)	0.56
PTT (sec)	40 (33–49)	40 (33–56)	43 (34–53)	0.68
Duration of hospitalization	20 (12–26)	29 (19–37)	15 (7–36)	0.051
MELD score after therapy	31 (26–33)	27 (24–34)	35 (25–39)	**0.03**
OPAL vs. Prometheus				ns
OPAL vs. SMT				ns
Prometheus vs. SMT				**0.03**
CLIF‐C ACLF score after therapy	47 (43–50)	44 (0–49)	49 (44–58)	**0.007**
OPAL vs. Prometheus				ns
OPAL vs. SMT				ns
Prometheus vs. SMT				**0.007**

Abbreviations: ACLF, Acute‐on‐chronic liver failure; ALT, alanine transaminase; AST, aspartate transaminase; AP, alkaline phosphatase; CLIF‐C, Chronic Liver Failure Consortium; GGT, gamma‐glutamyltransferase; LDH, lactate dehydrogenase; MELD, Model for End‐Stage Liver Disease; ns, not significant; PTT, partial thromboplastin time; SMT, standard medical treatment.

### 3.2 Alterations in Laboratory Values and Clinical Scores After OPAL Treatment

**FIGURE 1:**
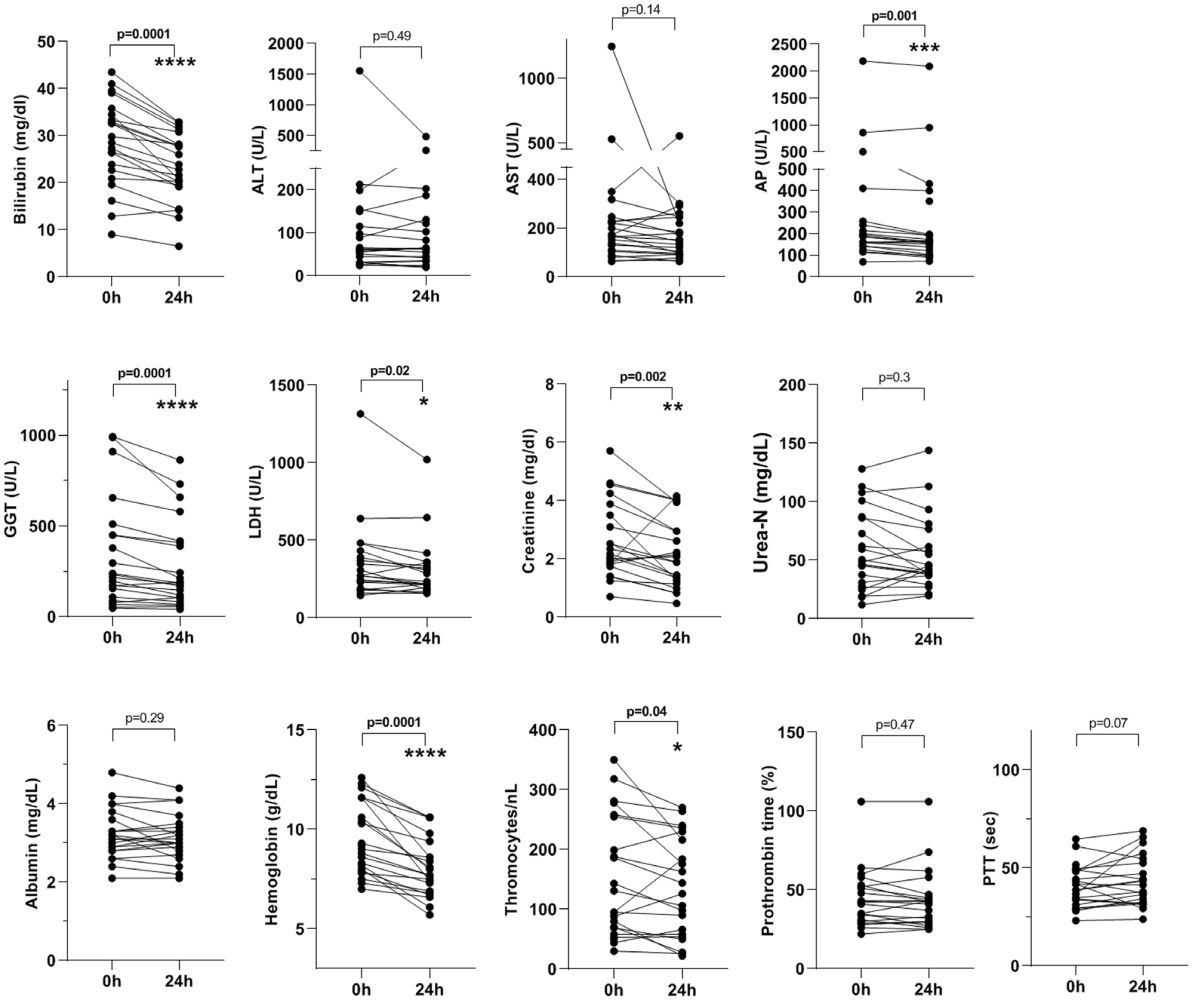
Changes in biochemical variables of 22 ACLF patients after the first session of treatment with OPAL. ACLF, Acute‐on‐chronic liver failure; ALT, alanine transaminase; AST, aspartate transaminase; AP, Alkaline phosphatase; GGT, gamma‐glutamyltransferase; LCH, lactate dehydrogenase; PTT, partial thromboplastin time.

**FIGURE 2:**
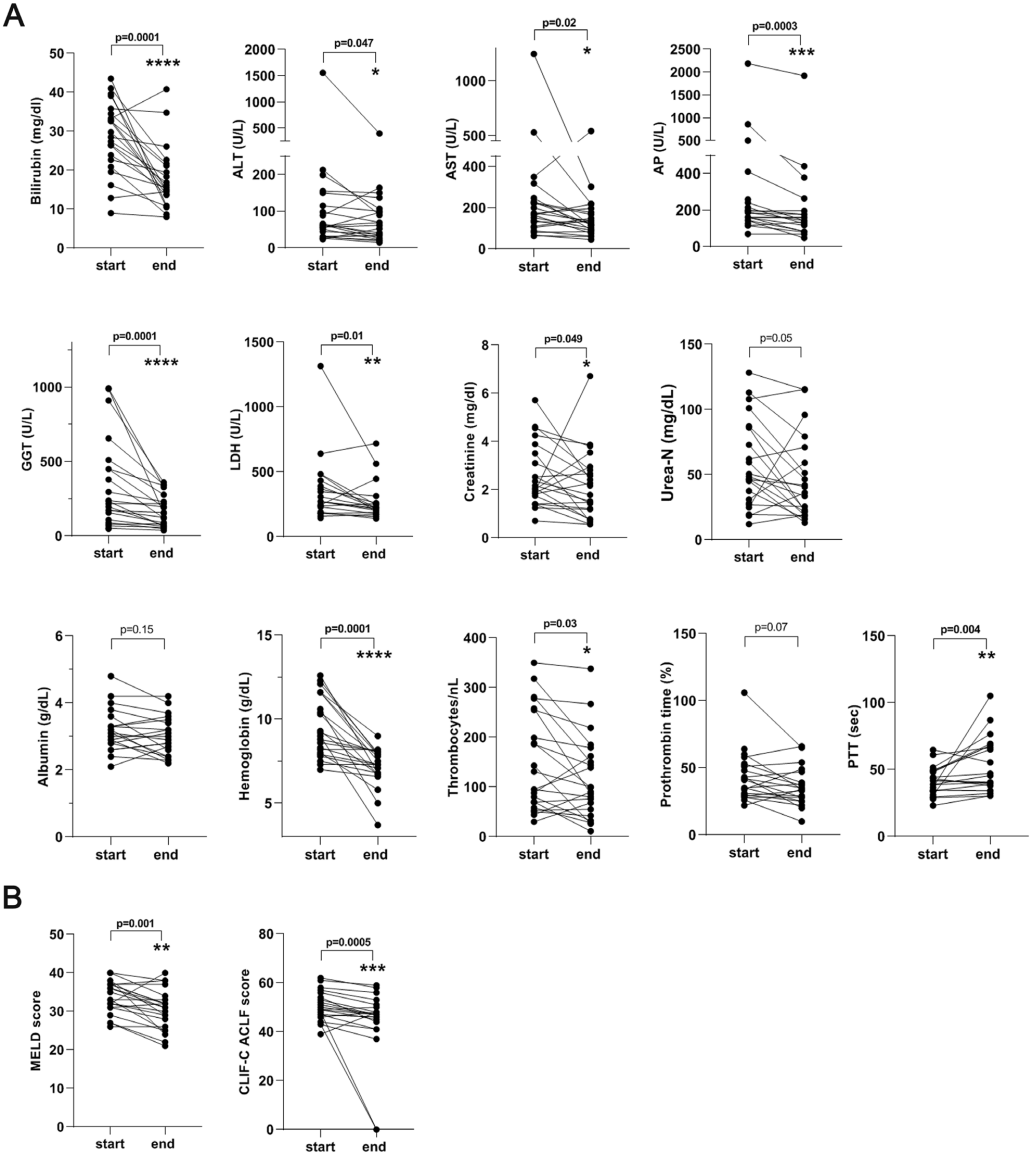
Changes in biochemical parameters (A) and clinical scoring (B) of 22 ACLF patients 24hours after the end of treatment with OPAL. ACLF, Acute‐on‐chronic liver failure; ALT, alanine transaminase; AST, aspartate transaminase; AP, alkaline phosphatase; CLIF‐C, Chronic Liver Failure Consortium; GGT, gamma‐glutamyltransferase; LDH, lactate dehydrogenase; MELD, model for end‐stage liver disease; PTT, partial thromboplastin time.

We assessed the changes in laboratory values 24 h after the first OPAL session (Figure 1) and after the completion of OPAL treatment (Figure 2). The first OPAL treatment session resulted in a statistically significant reduction in total serum bilirubin, serum creatinine, hemoglobin (*p* = 0.0001) levels, platelet counts, alkaline phosphatase (AP), gamma‐glutamyltransferase (GGT) and lactate dehydrogenase (LDH) (Figure 1). The complete OPAL treatment led to a statistically significant reduction in serum bilirubin and serum creatinine levels, transaminase activities, partial thromboplastin time, platelet counts, and hemoglobin values (Figure 2A).

Regarding risk stratification scores, we observed a significant amelioration of the MELD and CLIF‐C ACLF scores obtained at the time of admission to the hospital and those obtained 24 h after the termination of OPAL therapy (Figure 2B).

### 3.3 Changes in Laboratory Parameters Between Patients Treated With OPAL and Those Treated With Either Prometheus or SMT Plus Hemodialysis

As shown in Table 1, the comparison of the baseline characteristics between OPAL and Prometheus patients revealed no statistically significant differences between the two alternative treatment groups. Nevertheless, we observed a trend toward a higher number of ACLF cases occurring due to infection among patients treated with Prometheus than among those treated with OPAL (Table 1). On the other hand, the OPAL group tended to include more ACLF cases related to other etiologies besides the common reasons such as infection, bleeding, or alcohol abuse than the Prometheus group (Table 1). Regarding Table 2, blood flow was significantly lower in the OPAL group than in the Prometheus group, while baseline values of aspartate transaminase were statistically significantly higher among patients treated with OPAL than among patients treated with Prometheus. However, pretreatment values of bilirubin were comparable for both procedures (Table 2). However, comparing the OPAL and SMT groups, we observed that hepatorenal syndrome, ACLF Grade 1, and infections as causes of ACLF were more common in the SMT group (Table 1). Moreover, patients in the SMT group were significantly older with a higher CLIF‐C ACLF score at hospital admission (Table 2). Pretreatment serum bilirubin values were significantly lower in the SMT group (Table 2). Table S1 shows a comparison of the baseline characteristics of patients undergoing Prometheus dialysis and those treated with SMT.

**FIGURE 3:**
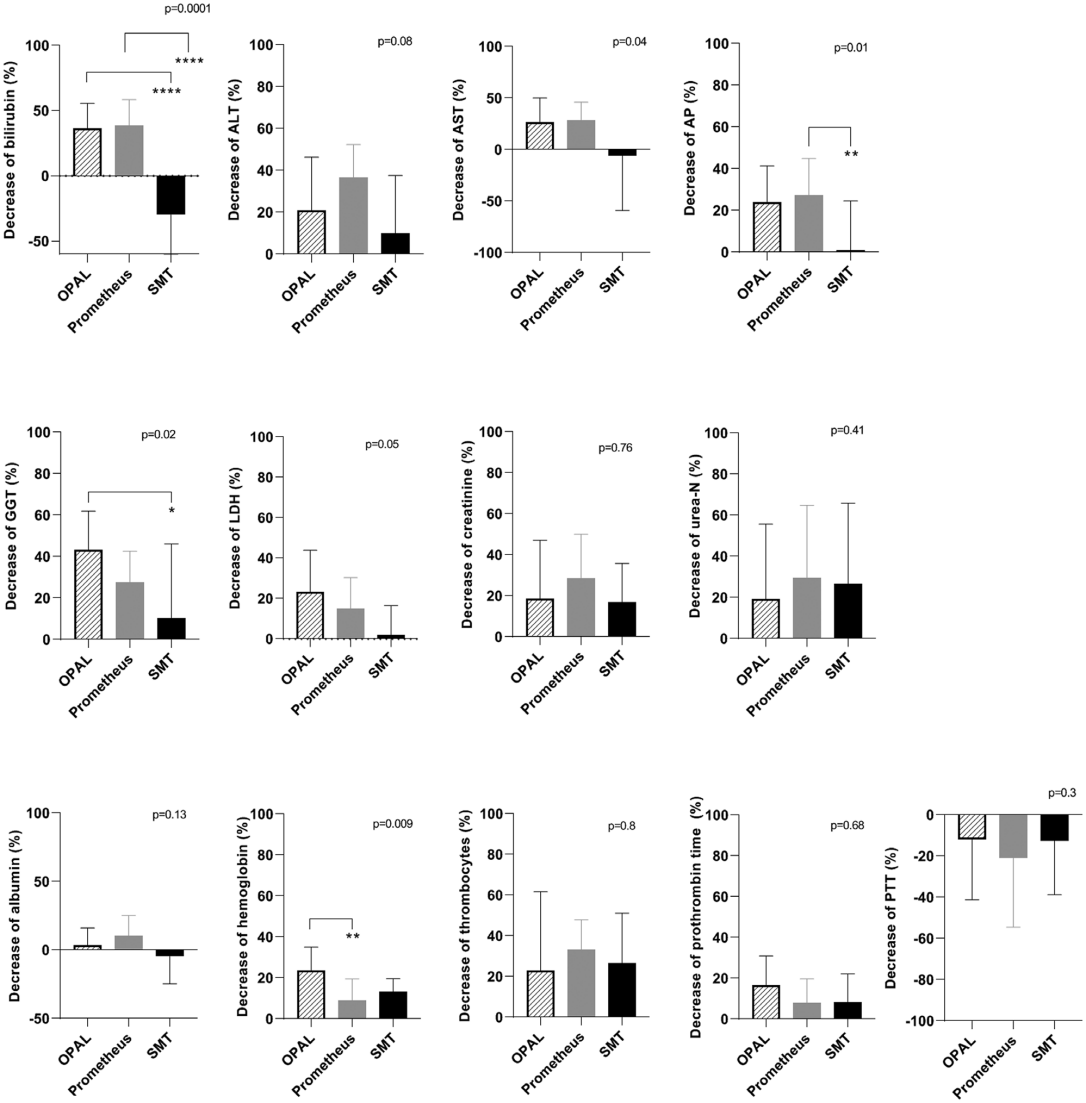
Relative percentage changes in laboratory values of ACLF patients 24 h after the end of treatment with OPAL (22 patients), Prometheus (41 patients), or SMT plus hemodialysis (24 patients). ACLF, Acute‐on‐chronic liver failure; ALT, alanine transaminase; AST, aspartate transaminase; AP, alkaline phosphatase; GGT, gamma‐glutamyltransferase; LDH, lactate dehydrogenase; PTT, partial thromboplastin time; and SMT, standard medical treatment.

With respect to the ability to clear bilirubin, we detected a significantly greater percentage reduction of bilirubin levels in the OPAL and Prometheus treatment groups than in the SMT group (Figure 3). The relative decrease in bilirubin concentrations in the OPAL group ranged from 13% to 75% in comparison to baseline values (Figure 3). Interestingly, OPAL therapy strongly reduced hemoglobin levels (Figure 3), with similar relative reduction rates in AST, ALT, and LDH activities and in prothrombin time, PTT, creatinine, urea‐N, and platelet values for all three procedures (Figure 3).

Furthermore, the first treatment session with the OPAL device resulted in significant augmentation of the systolic and diastolic blood pressure after treatment, suggesting positive effects of OPAL on stabilization of the hemodynamic situation in patients with ACLF (Table 4). Systolic and diastolic blood pressures did not significantly change under Prometheus and hemodialysis (Table 4). However, Prometheus was administered at the highest blood flow rate of 200 mL/min, whereas OPAL therapy was administered at the relatively lowest blood flow of 150 mL/min (Table 4).

### 3.4 Effect of OPAL, Prometheus, and SMT on Mortality and Clinical Scoring

**FIGURE 4:**
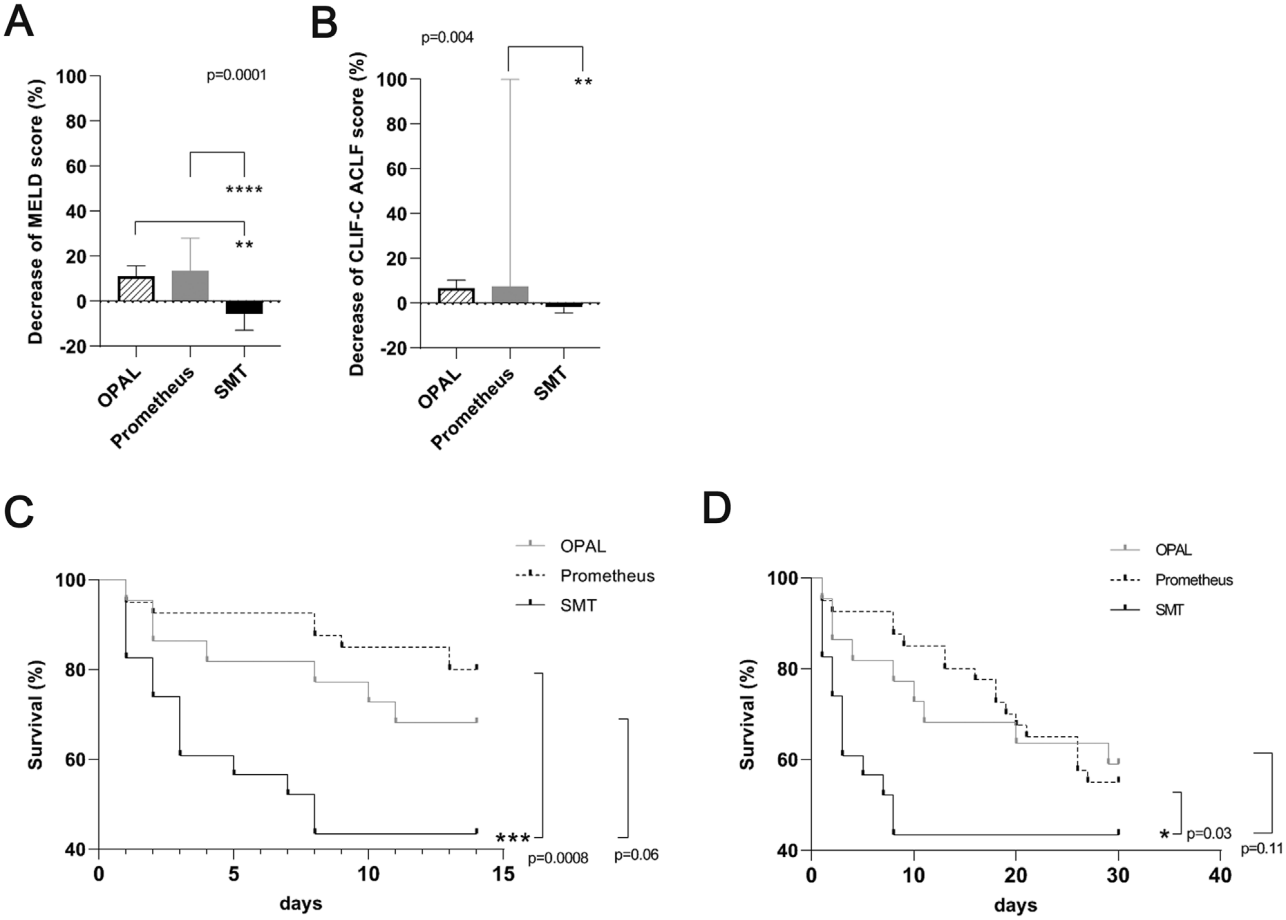
Effect of treatment with various liver support procedures on clinical scoring and overall mortality rates of ACLF patients. (A‐B) Relative percentage changes in MELD (A) and CLIF‐C ACLF (B) scores 24 h after the end of ACLF treatment with either OPAL, Prometheus, or SMT plus hemodialysis. (C) Short‐term 14‐day mortality rates of ACLF patients receiving OPAL (22 patients), Prometheus (41 patients), or SMT plus hemodialysis (24 patients). (D) 30‐day mortality rates of ACLF patients treated with OPAL, Prometheus, or SMT plus hemodialysis. ACLF, Acute‐on‐chronic liver failure; CLIF‐C, Chronic Liver Failure Consortium; MELD, Model for End‐Stage Liver Disease; SMT, standard medical treatment.

The relative decline in the MELD score was significantly greater in the OPAL and Prometheus groups than in the SMT group (Figure 4A). Comparison of OPAL with Prometheus dialysis found that the two devices had achieved similar MELD score reductions at the end of therapy (Figure 4A). We found a difference in the relative reduction of the CLIF‐C ACLF score only between the Prometheus dialysis group and the SMT group (Figure 4B). As expected, the highest posttreatment MELD and CLIF‐C ACLF scores were seen for the SMT group (Table 2).

Prometheus yielded significantly lower short‐term mortality rates (deaths occurring within the first 14 days after the end of therapy, Figure 4C) and 30‐day mortality rates (Figure 4D) for ACLF patients than did SMT. Patients treated with OPAL also tended to have better survival rates than did those receiving SMT (14‐day mortality OPAL vs. SMT *p* = 0.06; 30‐day mortality OPAL vs. SMT *p* = 0.11; Figure 4C,D). The survival rates did not differ significantly between the OPAL and Prometheus groups (Figure 4C,D). The 14‐day and 30‐day survival rates of ACLF patients treated with OPAL or Prometheus dialysis continued to be better than those of patients treated with SMT even after patients who had no evidence of renal impairment were excluded from the OPAL and Prometheus groups (Figure S1).

Differentiating between patients with ACLF Grade 1 and 2 for each procedure group showed that the 14‐day mortality rate was not related to the ACLF grade (Figure S2A), but patients with ACLF Grade 2 who were treated with Prometheus dialysis tended to have higher 30‐day mortality rates than patients with ACLF Grade 1 (*p* = 0.09; Figure S2B).

The frequency of bleeding events, predominately gastrointestinal bleeding or bleeding from the puncture site of the dialysis catheter, during extracorporeal treatment was similar for all three treatment options. The infection rate was also comparable between the three therapy approaches.

### 3.5 Course of Kidney and Liver Function After Treatment With OPAL and Prometheus Versus SMT

Considering the development of kidney function after the completion of ECLS therapies or SMT, we observed that ACLF patients who underwent SMT required continuation of renal replacement significantly more frequently than those who were previously treated with OPAL [10/22 (46%) vs. 21/24 (88%), *p* = 0.002; Table 3]. No differences were seen between the OPAL and Prometheus devices (Table 3). Separate analysis of ACLF patients who survived longer than 30 days after the end of ECLS or SMT indicated that the number of ACLF patients who developed a new onset chronic end‐stage kidney disease requiring dialysis (CKDG5D) was significantly higher in the SMT group than in the OPAL group [6/10 (60%) vs. 1/13 (8%), *p* = 0.007, Table 3]. Comparison between the OPAL and Prometheus groups also revealed a trend toward more CKDG5D cases among survivors treated with Prometheus than among survivors treated with OPAL [7/22 (26%) vs. 1/13 (8%), p = 0.007, Table 3]. The highest rate of development of renal function impairment according to chronic kidney disease grades 3–5 in survivors was detected in the SMT group (70%), followed by Prometheus (64%) and OPAL (46%) (Table 3).

**TABLE 3:** Comparison of outcome variables of ACLF patients after treatment with OPAL (22 patients), Prometheus (41 patients), or SMT plus hemodialysis (24 patients).

Variable	OPAL *n* = 22	SMT *n* = 24	RR (CI)	*p* value	OPAL *n* = 22	Prometheus *n* = 41	RR (CI)	*p* value
Death within 14 days after treatment, *n* (%)	7 (32)	14 (58)	0.55 (0.3–1.1)	*0.07*	7 (32)	9 (22)	1.45 (0.6–3.3)	0.39
Death within 30 days after treatment, *n* (%)	9 (41)	14 (58)	0.7 (0.4–1.3)	0.24	9 (41)	19 (46)	0.88 (0.5–1.6)	0.68
Transfer to ICU, *n* (%)	2 (9)	5 (21)	0.44 (0.1–1.7)	0.27	2 (9)	2 (5)	1.86 (0.3–10.0)	0.51
Need to continue RRT, (%)	10 (46)	21 (88)	0.52 (0.3–0.8)	**0.002**	10 (46)	24 (59)	0.78 (0.4–1.3)	0.32
New onset CKDG5D among survivors, (%)	1/13 (8)	6/10 (60)	0.13 (0.02–0.6)	**0.007**	1/13 (8)	7/22 (26)	0.21 (0.04–1.1)	*0.06*
New onset CKDG3‐5 among survivors, (%)	6/13 (46)	7/10 (70)	0.66 (0.3–1.4)	0.25	6/13 (46)	14/22 (64)	0.73 (0.3–1.3)	0.31
MELD score at 3 days after the end of treatment, (IQR)	32 (31–38)	29 (20–32)		**0.04**	32 (31–38)	26 (20–32)		**0.006**
Percentage reduction of MELD score at 3 days after the end of treatment, (IQR)	2.5 (−8.5–12.5)	12.3 (−5.6–31.6)		0.32	2.5 (−8.5–12.5)	16.7 (4.2–28.9)		**0.01**
INR at 3 days after the end of treatment, (IQR)	1.8 (1.5–2.6)	1.3 (1.2–1.8)		*0.07*	1.8 (1.5–2.6)	1.5 (1.3–1.8)		**0.04**
Percentage reduction of INR at 3 days after the end of treatment, (IQR)	−13.1 (−32.5–5.6)	8.6 (−10.6–19.2)		0.13		−2.7)‐ 7.1–6.9)		0.14
New hospitalizations, *n* (%)	1 (5)	2 (8)	0.6 (0.1–3.9)	0.55	1 (5)	6 (15)	0.31 (0.1–1.8)	0.22
Liver transplant after treatment, *n* (%)	1 (5)	0 (0)	Infinity (0.3–infinity)	0.29	1 (5)	4 (10)	0.47 (0.1–2.8)	0.47
Bleeding during treatment, *n* (%)	7 (32)	7(29)	1.1 (0.5–2.6)	0.85	7 (32)	8(20)	1.63 (0.7–3.8)	0.27
Infection during treatment, *n* (%)	3 (14)	3 (13)	1.1 (0.3–4.3)	0.91	3 (14)	3 (7)	1.86 (0.5–7.5)	0.42

Abbreviations: ACLF, Acute‐on‐chronic liver failure; CI, confidence interval; CKDG, chronic kidney disease grade; CKDG5D, end‐stage chronic kidney disease requiring dialysis; ICU, intensive care unit; INR, international normalized ratio; IQR, interquartile range; MELD, Model for End‐Stage Liver Disease; OPAL, open albumin dialysis; RR, relative risk; RRT, renal replacement therapy; SMT, standard medical treatment.

**TABLE 4:** Alterations in systolic and diastolic blood pressure and the need for pharmaceutical measures for circulatory support during the first session of OPAL versus Prometheus dialysis versus SMT plus hemodialysis.

	Median systolic blood pressure pretreatment (IQR)	Median systolic blood pressure posttreatment (IQR)	*p* value	Median diastolic blood pressure pretreatment (IQR)	Median diastolic blood pressure posttreatment (IQR)	*p* value	Use of human albumin during treatment, *n* (%)	Use of terlipressin during treatment, *n* (%)	Use of intravenous volume therapy during treatment, *n* (%)
**OPAL, *n* = 22**	108 (101–116)	118 (107–140)	**0.0002**	62 (58–71)	70 (60–78)	**0.02**	2 (9)	4 (18)	0 (0)
**Prometheus, *n* = 41**	113 (100–127)	113 (93–123)	0.26	61 (55–69)	62 (54–67)	0.66	1 (2)	3 (7)	18 (44)
**SMT, *n* = 24**	108 (94–131)	96 (93–125)	0.32	57 (50–70)	56 (51–65)	0.29	7 (29)	5 (21)	0 (0)

Abbreviations: IQR, Interquartile range; OPAL, open albumin dialysis; SMT, standard medical treatment.

All ACLF patients who were treated with both ECLS devices and SMT including hemodialysis were on the normal ward and did not need intensive care unit management during treatment. In order to assess hemodynamic stability after the application of ECLS devices versus SMT including hemodialysis, we analyzed the occurrence of transfer to the intensive care unit after the end of ECLS or SMT. As illustrated in Table 3, we observed no significant differences between the three treatment groups.

Severe hepatic encephalopathy appeared in a few patients among the three treatment groups. Among ACLF patients treated with OPAL, hepatic encephalopathy grades 2–3 at the start of OPAL treatment was present in three patients, of whom only one patient recovered after the completion of OPAL. Similarly, only three patients in the SMT group experienced hepatic encephalopathy grades 2–3 at the beginning, and hepatic encephalopathy symptoms resolved in one patient. In the Prometheus group, hepatic encephalopathy grades 2–3 was observed in seven patients at baseline and disappeared after the end of treatment in three patients.

In order to evaluate the further development of liver function after the end of corresponding treatment, we took into account MELD score and international normalized ratio (INR) values obtained 3 days after the end of treatment (Table 3). MELD scores determined in the SMT group and Prometheus group were significantly lower compared to the OPAL group (Table 3). The percentage reduction of the MELD score on Day 3 after the end of treatment in relation to the baseline MELD score was significantly higher in the Prometheus group than in the OPAL group (Table 3). INR values quantified 3 days after the completion of treatment were lower in the Prometheus group and SMT group in comparison to the OPAL group (Table 3). However, we suppose that the results achieved for the SMT group are strongly influenced by selection bias because most ACLF patients in this group needed continuation of hemodialysis; accordingly, MELD score and INR were assessed only in a minority of patients who recovered from hepatorenal syndrome and in whom SMT including hemodialysis was terminated.

### 3.6 The Degree of Relative Reduction in Serum Bilirubin Levels After OPAL or Prometheus Predicts Mortality Rates and Clinical Scoring

**FIGURE 5:**
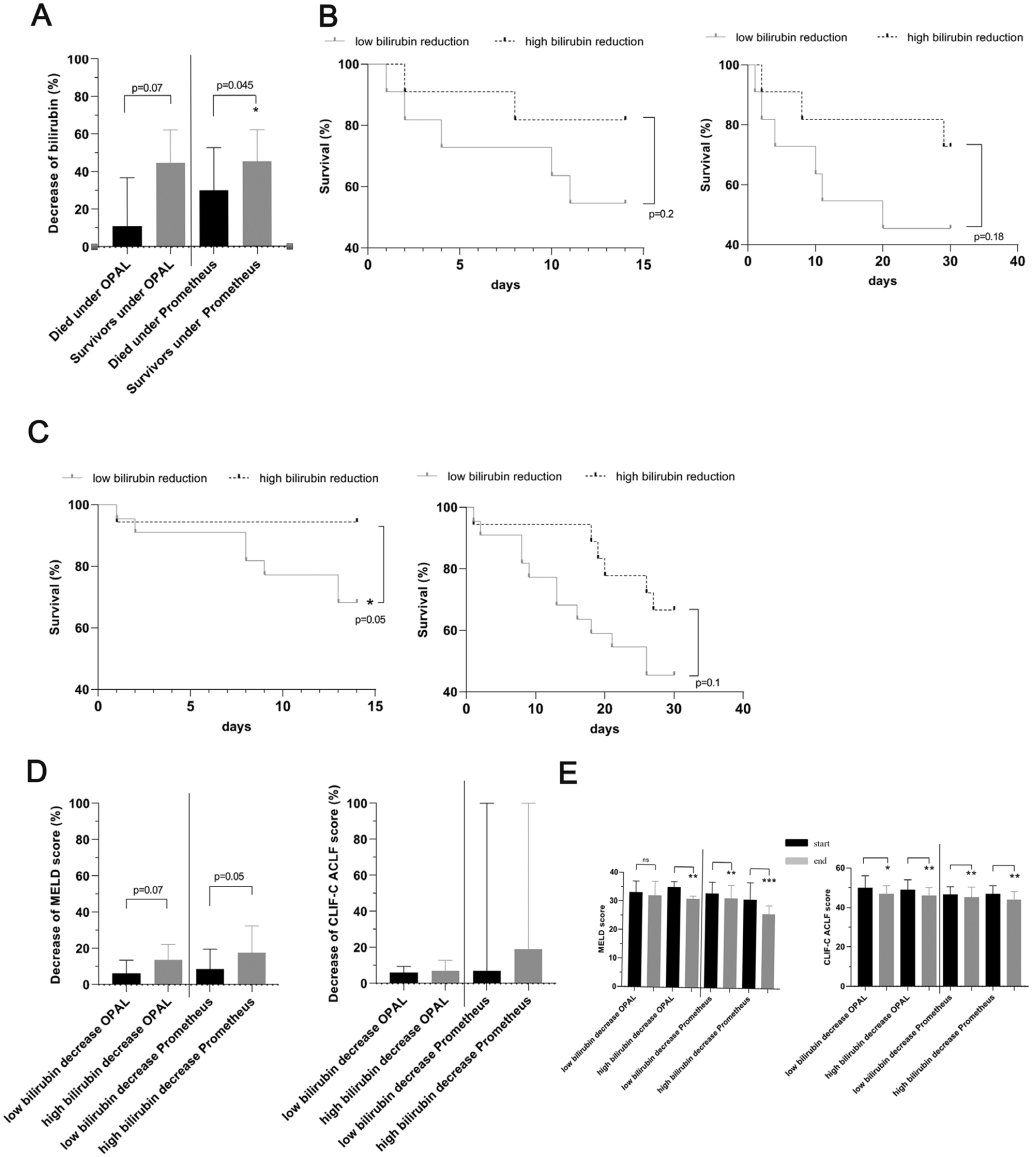
Overall mortality rates and clinical scoring of ACLF patients in relation to the median relative serum bilirubin reduction achieved by OPAL or Prometheus. (A) Assessment of the relative percentage change in bilirubin concentration among ACLF patients who survived or died under OPAL therapy or Prometheus dialysis. (B) 14‐day and 30‐day mortality rates in dependence on relative serum bilirubin reduction among ACLF patients treated with OPAL. (C) 14‐day and 30‐day mortality rates in dependence on relative serum bilirubin reduction among ACLF patients treated with Prometheus. (D) Relative decrease in MELD and CLIF‐C ACLF scores in dependence on relative serum bilirubin reduction among ACLF patients treated with OPAL or Prometheus. (E) Comparison of the pretreatment and posttreatment MELD and CLIF‐C ACLF scores among ACLF patients treated with the OPAL or Prometheus device in relation to the relative percentage reduction in serum bilirubin levels achieved with the indicated liver support therapy. *p = 0.05; **p = 0.01; ***p = 0.0001. ACLF, Acute‐on‐chronic liver failure; CLIF‐C, Chronic Liver Failure Consortium; MELD, model for end‐stage liver disease; OPAL, open albumin dialysis.

Furthermore, we observed that ACLF patients who died after treatment with Prometheus exhibited a statistically significant lower relative decrease in bilirubin levels than those who survived (Figure 5A). In the case of those ACLF patients in whom OPAL was applied, there was a clear trend toward higher relative reduction of bilirubin due to OPAL among survivors compared to nonsurvivors, which did not reach statistical significance (Figure 5A). Next, for both OPAL and Prometheus procedures, we defined two groups with a treatment‐related percentage reduction of total serum bilirubin either above or below the median bilirubin reduction rate. Figure 5 demonstrates that the 14‐day and 30‐day mortality rates of ACLF patients treated with either OPAL (Figure 5B) or Prometheus (Figure 5C) were lower for patients with a bilirubin decrease above the median reduction rate than for patients with a bilirubin decrease below the median.

Both OPAL and the Prometheus device were associated with a trend toward a greater relative reduction of the MELD score at the end of treatment for the patients with a relative bilirubin decrease above the median as cut‐off (for OPAL 6.3% vs. 13.8% *p* = 0.07; for Prometheus 8.8% vs. 17.7% *p* = 0.051; Figure 5D). We did not find any relative reduction in the CLIF‐C ACLF score after either of these therapies (Figure 5D). Strikingly, the OPAL procedure had a statistically significant effect on the MELD score only among those ACLF patients with a strong relative bilirubin reduction above the cut‐off (Figure 5E).

## 4 Discussion

The present study was aimed at investigating the feasibility of OPAL therapy for ACLF patients and also at comparing the effect of OPAL with that of other ECLS and SMT on clinical scoring and overall mortality. With regard to the detoxification performance, ECLS, Prometheus, and OPAL allowed an efficient removal of bilirubin superior to hemodialysis and a creatinine removal that was comparable with the performance of hemodialysis. The most experience was gained with the predecessor of the OPAL, MARS, showing a sufficient purification of bilirubin in ACLF [5, 7, 14, 15]. Krisper et al. conducted a cross‐over study comparing MARS and Prometheus and identified a better performance for Prometheus in the removal of conjugated and unconjugated bilirubin [16]. Other studies reported that MARS was able to effectively reduce creatinine in the blood of ACLF patients [5, 7, 14, 15], whereas Prometheus was shown not to modify creatinine levels in some studies [16, 17, 18, 19]. In contrast to the above‐mentioned observations, our study revealed that Prometheus and OPAL both exhibited similar abilities in the elimination of creatinine that were comparable with the performance of classical hemodialysis included in the SMT in our studied patient cohorts. Sommerfeld and colleagues conducted a retrospective single‐center study at Jena University Hospital and compared four different ECLS devices including MARS, OPAL, advanced organ support (ADVOS), and single‐pass albumin dialysis in 96 critically ill intensive care unit patients with ALF, ACLF, or liver transplant failure induced acute liver dysfunction [7]. In this study, several patients were treated with different ECLS systems on different days between 2015 and 2021 [7]. Analysis of available sessions performed with the four ECLS devices revealed similar bilirubin elimination rates for all four ECLS modes, whereas removal of ammonia was significantly better using OPAL and ADVOS [7]. Clearance of creatinine and urea after 64 sessions of OPAL was comparable with clearance rates achieved after 167 cycles of ADVOS and 54 cycles of MARS [7]. The percental bilirubin elimination rate of 16% yielded after repeated sessions of OPAL was lower than in our study, while the elimination rates of creatinine and urea obtained after 64 OPAL sessions were in line with our results [7]. No significant differences in in‐hospital mortality rates and clinical scores obtained at the end of ECLS such as MELD, APACHE II, SOFA, and SAPS II were detected when comparing the four ECLS devices [7]. Another prospective multicenter study by Stange et al. investigated the feasibility of ECLS with OPAL in ACLF patients in comparison to MARS [20]. Overall, 30 ACLF patients were randomly treated with OPAL and MARS in a cross‐over design [20]. Reduction of bile acids and improvement of albumin binding capacity to bile acids were higher after OPAL than after MARS application [20]. In contrast to our research work, the authors did not report any data on the improvement of clinical scores or survival in the study [20].

We saw beneficial effects of the first session of OPAL on hemodynamics with a significant increase in systolic and diastolic blood pressure in ACLF patients, while the first single session of ECLS with Prometheus did not impact blood pressure levels in ACLF patients in our study. These observations are in concordance with prospective data presented by Dethloff et al. demonstrating that a single 6‐h application of MARS was associated with a relevant elevation in arterial blood pressure in eight patients having decompensation of liver cirrhosis, whereas 6‐h treatment with Prometheus did not influence blood pressure in the other eight patients [21]. In contrast to our study, blood pressure was assessed with a Swan‐Ganz catheter [21]. A former randomized controlled study by Iarustovskiĭ et al. also supports our findings on the improvement of hemodynamics upon albumin dialysis [22]. Amelioration of hemodynamic instability with an increase in mean arterial blood pressure was significantly greater in nine patients who were assigned to the MARS group and treated with one to two sessions of MARS compared to 17 patients who received up to 14 cycles of Prometheus [22]. However, the study population of our study substantially differed from the patients enrolled in the study of Iarustovskiĭ et al., including elderly ALF patients who underwent cardiac surgery [22].

In the current study, ACLF patients treated with Prometheus dialysis experienced better 14‐day and 30‐day survival rates than did patients in the SMT group. Our data on Prometheus contradict the results presented by Kribben et al.; their study found no differences in survival rates between ACLF patients treated with Prometheus and those treated with SMT [23], which is in line with several other research groups [5, 6, 14]. Some subgroup analyses showed that Prometheus had favorable effects on the survival of ACLF patients with severe disease referred to as MELD scores higher than 30 points [23]. In our study, the Prometheus group exhibited significantly lower CLIF‐C ACLF scores at baseline and was significantly younger than the patients treated with SMT; these characteristics may have contributed to the fact that Prometheus was superior to SMT with regard to 14‐day and 30‐day survival rates. Although Prometheus has disappeared from the European market, it is still available and widely used in other countries of the world, like in Asian countries [11]. Thus, results on Prometheus provided by our study might be of great relevance for users in several other countries outside of Europe.

Meanwhile, patients in the OPAL group exhibited a trend toward better 14‐day and 30‐day survival rates than did patients in the SMT group, but the difference did not reach statistical significance. This lack of a significant effect may be partly explained by the fact that patients in the OPAL group (Table 1) exhibited higher ACLF grades than did patients in the SMT group, among whom ACLF Grade 1 predominated, even though baseline CLIF‐C ACLF and MELD scores did not differ between the two groups (Table 2).

The occurrence of acute kidney failure among ACLF patients is mainly due to hepatorenal syndrome, which is well known to aggravate the prognosis of ACLF and to be associated with poor overall survival rates [24]. Nonetheless, the results of our survival analysis including only patients with hepatorenal syndrome in the OPAL and Prometheus groups were similar to those obtained in a previous analysis that took into account all ACLF patients regardless of the appearance of kidney injury.

In general, the results obtained from previous clinical studies on the association of artificial liver support systems with mortality rates for ACLF patients are often conflicting. Retrospective study design, small number of patients, and different definitions of ACLF hamper external validation and comparability. The study populations, as well as reported treatment protocols, show high heterogeneity; for example, due to combining data from ACLF, acute decompensation, and acute liver failure.

In this study, the use of ECLS resulted in better clinical scoring values than did the use of SMT. Most studies concentrated on overall and transplant‐free survival as leading clinical outcomes, but improvement in clinical scores was often not examined. However, improvement in prognostic scores, such as the MELD score or the CLIF‐C ACLF score, due to the reduction in bilirubin values does not necessarily reflect restored liver function and therefore should be interpreted with caution.

Our study has several limitations, including its retrospective design and a relatively small sample size, particularly in the OPAL and the SMT groups. The fact that allocation to OPAL at our center was not random may have caused serious selection bias. Furthermore, including only ACLF patients who had been exclusively treated with intermittent hemodialysis in the SMT group may also have produced selection bias. ACLF patients who received SMT but required continuous hemodialysis (mostly because of hypotension) and who were predisposed to a poorer survival prognosis were excluded from the analysis; this fact may have led to an underestimation of the favorable effects of OPAL or Prometheus liver support devices. Differences in certain baseline characteristics between the groups may also have biased the subsequent results. In particular, results deriving from the comparison of the two ECLS treatments with SMT including hemodialysis should be interpreted with caution because of the high heterogeneity of the groups. Those ACLF patients who were allocated to the SMT group were older, suffered more often from hepatorenal syndrome and multiorgan failure, and displayed a tendency toward higher baseline CLIF‐C ACLF scores than ACLF patients in the OPAL group, reflecting that patients belonging to the SMT group were sicker and incorporated an association with higher mortality than patients from the OPAL group, which might have contributed to the better performance of the OPAL group in our study in comparison to the SMT group. Moreover, patients in the Prometheus group exhibited significantly lower CLIF‐C ACLF scores at baseline, experienced less frequently multiorgan failure and hepatorenal syndrome, and were significantly younger than the patients treated with SMT; these characteristics may have contributed to the fact that Prometheus was superior to SMT with regard to 14‐day and 30‐day survival rates. The use of SMT alone is required for stabilizing patients with a low ACLF grade and providing them with the chance of a resolution. However, patients exhibiting ACLF Grade 2 or 3 are less likely to recover under SMT and may profit more from the administration of an artificial liver support device. However, this was a pilot study exploring for the first time the efficacy and safety of OPAL for adult patients with ACLF.

Our observations of the adverse effects associated with OPAL therapy agree with those previously reported for the MARS system: bleeding at the catheter site and thrombocytopenia [5, 14]. In contrast, we observed a significantly stronger reduction in hemoglobin levels after OPAL treatment than after Prometheus. Thus, medical professionals should be cautiously aware of this possible complication of OPAL and should consider that transfusion may be required.

The initial levels of bilirubin at the initiation of therapy have prognostic relevance for ACLF patients [25]. We found that the mild relative reduction of bilirubin levels associated with both artificial liver support methods (OPAL and Prometheus) led to increased mortality rates and little improvement in the clinical scores of ACLF patients. Thus, we propose that the degree of relative bilirubin reduction associated with each liver support system may be helpful in finding candidates with an unfavorable clinical course of ACLF and with an elevated risk of mortality. The continuation of artificial liver support therapy should be carefully reconsidered for ACLF patients who did not experience adequate bilirubin reduction despite ongoing artificial liver support.

Currently, liver transplant as a treatment option for ACLF patients is not easily accessible and demands lifelong immunosuppression. Our study highlights the potential benefits of ECLS systems as an additional option for the optimal treatment of ACLF patients. However, the decision as to which currently available ECLS methods should be used for which ACLF patients remains a challenge for clinicians. Furthermore, patients with a MELD score lying above 28 in the absence of more than two organ failures or overt sepsis are prioritized for early liver transplant [26]. However, the usage of ECLS devices such as OPAL or Prometheus enables a strong reduction of the MELD score [26]. The decrease of the MELD score provoked by the application of such ECLS systems in potentially eligible liver transplant candidates with ACLF prior to transplant might provide disadvantages to these patients in competing for organs by reducing their priority on the waiting list for liver transplant [27]. This circumstance raises concern about the need to adapt regulations for liver transplants with regard to those transplant centers where ECLS devices are available and commonly used.

Additional research, including prospective randomized clinical trials, should be done to determine the appropriate liver support device and the correct initiation time of liver support therapy for various subgroups of ACLF patients that might bring us closer to personalized therapy for ACLF.

## Author Contributions

Concept/design, **J.F.K**., **A.Z**., **A.K**., **M.J**., **H.H.S**., and **B.T**.; data analysis/interpretation, **J.F.K**., **A.Z**., and **M.J**.; drafting article, **J.F.K**. and **A.Z**.; critical revision of article, **A.K**., **M.J**., **H.H.S**., **B.T**., **A.S**., and **J.R.A**.; statistics, **J.F.K**. and **A.L**.; funding secured by **A.K**. and **H.H.S**.; data collection, **J.F.K**., **A.L**., and **A.Z**.; supervision, **J.F.K**.; and project administration, **J.F.K**., **A.Z**., **M.J**., **A.S**., and **J.R.A**.

## Conflicts of Interest

The authors declare no conflicts of interest.

## Supporting information


**Figure S1.** Effect of treatment with various liver support procedures on overall mortality, considering only ACLF patients with acute kidney injury. (A) Short‐term 14‐day mortality rates comparing OPAL therapy with Prometheus therapy and with SMT plus hemodialysis. (B) Thirty‐day mortality rates comparing OPAL therapy with Prometheus therapy and with SMT plus hemodialysis.
ACLF, acute‐on‐chronic liver failure; OPAL, open albumin dialysis; SMT, standard medical treatment.

**Figure S2.** Effect of treatment with various liver support procedures on overall mortality in dependence on ACLF grade. (A) Short‐term 14‐day mortality rates comparing OPAL therapy with Prometheus therapy and with SMT plus hemodialysis. (B) Thirty‐day mortality rates comparing OPAL therapy with Prometheus therapy and with SMT plus hemodialysis.
ACLF, acute‐on‐chronic liver failure; OPAL, open albumin dialysis; SMT, standard medical treatment.

**Table S1.** Comparison of baseline characteristics of 41 ACLF patients treated with Prometheus and 24 ACLF patients treated with SMT plus hemodialysis.

## References

[1] G. Perricone , T. Artzner , E. de Martin , R. Jalan , J. Wendon , and M. Carbone , “Intensive Care Management of Acute‐On‐Chronic Liver Failure,” Intensive Care Medicine 49 (2023): 903–921. 37552333 10.1007/s00134-023-07149-x

[2] R. Moreau , R. Jalan , P. Gines , et al., “Acute‐On‐ Chronic Liver Failure Is a Distinct Syndrome That Develops in Patients With Acute Decompensation of Cirrhosis,” Gastroenterology 144, no. 7 (2013): 1426–1437, 10.1053/j.gastro.2013.02.042. 23474284

[3] S. Piano , N. Mahmud , P. Caraceni , M. Tonon , and R. P. Mookerjee , “Mechanisms and Treatment Approaches for ACLF,” Liver International 45, no. 3 (2025): e15733, 10.1111/liv.15733. 37715608 PMC12036731

[4] M. K. Nadim , F. Durand , J. A. Kellum , et al., “Management of the Critically Ill Patient With Cirrhosis: A Multidisciplinary Perspective,” Journal of Hepatology 64, no. 3 (2016): 717–735, 10.1016/j.jhep.2015.10.019. 26519602

[5] P. Papamichalis , K. G. Oikonomou , A. Valsamaki , et al., “Liver Replacement Therapy With Extracorporeal Blood Purification Techniques Current Knowledge and Future Directions,” World Journal of Clinical Cases 11, no. 17 (2023): 3932–3948, 10.12998/wjcc.v11.i17.3932. 37388799 PMC10303607

[6] R. Tandon and S. Froghi , “Artificial Liver Support Systems,” Journal of Gastroenterology and Hepatology 36, no. 5 (2021): 1164–1179. 32918840 10.1111/jgh.15255

[7] O. Sommerfeld , C. Neumann , J. Becker , et al., “Extracorporeal Albumin Dialysis in Critically Ill Patients With Liver Failure: Comparison of Four Different Devices‐ A Retrospective Analysis,” International Journal of Artificial Organs 46, no. 8–9 (2023): 481–491. 37609875 10.1177/03913988231191952 PMC10483887

[8] S. R. Mitzner , “Extracorporeal Liver Support‐Albumin Dialysis With the Molecular Adsorbent Recirculating System (MARS),” Annals of Hepatology 10, no. Suppl 1 (2011): S21–S28. 21566251

[9] E. Soo , A. Sanders , K. Heckert , T. Vinke , F. Schaefer , and C. P. Schmitt , “Comparison of Two Different Modes of Molecular Adsorbent Recycling Systems for Liver Dialysis,” Pediatric Nephrology Berlin Germany 31, no. 11 (2016): 2171–2174. 27394132 10.1007/s00467-016-3451-0

[10] K. Rifai , T. Ernst , U. Kretschmer , et al., “The Prometheus Device for Extracorporeal Support of Combined Liver and Renal Failure,” Blood Purification 23, no. 4 (2005): 298–302. 15980619 10.1159/000086552

[11] J. Dong , L. Huang , C. Li , B. Wu , X. Yang , and Y. Ge , “Fractionated Plasma Separation and Adsorption Integrated With Continuous Veno‐Venous Hemofiltration in Patients With Acute Liver Failure: A Single Center Experience From China,” Journal of Clinical Apheresis 39, no. 1 (2024): e22100. 37986652 10.1002/jca.22100

[12] P. Angeli , P. Ginès , F. Wong , et al., “Diagnosis and Management of Acute Kidney Injury in Patients With Cirrhosis: Revised Consensus Recommendations of the International Club of Ascites,” Journal of Hepatology 62, no. 4 (2015): 968–974, 10.1016/j.jhep.2014.12.029. 25638527

[13] P. S. Kamath , R. H. Wiesner , M. Malinchoc , et al., “A Model to Predict Survival in Patients With End‐Stage Liver Disease,” Hepatology 33, no. 2 (2001): 464–470, 10.1053/jhep.2001.22172. 11172350

[14] H. U. Gerth , M. Pohlen , G. Thölking , et al., “Molecular Adsorbent Recirculating System Can Reduce Short‐Term Mortality Among Patients With Acute‐On‐Chronic Liver Failure—A Retrospective Analysis,” Critical Care Medicine 45, no. 10 (2017): 1616–1624, 10.1097/CCM.0000000000002562. 28640024 PMC5598913

[15] K. Ocskay , A. Kanjo , N. Gede , et al., “Uncertainty in the Impact of Liver Support Systems in Acute‐On‐Chronic Liver Failure: A Systematic Review and Network Meta‐Analysis,” Annals of Intensive Care 11, no. 1 (2021): 10. 33462764 10.1186/s13613-020-00795-0 PMC7813174

[16] P. Krisper , V. Stadlbauer , and R. E. Stauber , “Clearing of Toxic Substances: Are There Differences Between the Available Liver Support Devices?,” Liver International 31, no. Suppl 3 (2011): 5–8. 21824275 10.1111/j.1478-3231.2011.02588.x

[17] P. Evenepoel , W. Laleman , A. Wilmer , et al., “Prometheus Versus Molecular Adsorbents Recirculating System: Comparison of Efficiency in Two Different Liver Detoxification Devices,” Artificial Organs 30, no. 4 (2006): 276–284. 16643386 10.1111/j.1525-1594.2006.00215.x

[18] A. Santoro , S. Faenza , E. Mancini , et al., “Prometheus System: A Technological Support in Liver Failure,” Transplantation Proceedings 38, no. 4 (2006): 1078–1082. 16757270 10.1016/j.transproceed.2006.02.017

[19] M. Grodzicki , M. Kotulski , D. Leonowicz , K. Zieniewicz , and M. Krawczyk , “Results of Treatment of Acute Liver Failure Patients With Use of the Prometheus FPSA System,” Transplantation Proceedings 41, no. 8 (2009): 3079–3081. 19857681 10.1016/j.transproceed.2009.08.024

[20] J. Stange , C. Sponholz , A. Kortgen , H. H. J. Schmidt , M. Dollinger , and T. I. Hassanein , “Open Albumin Dialysis (OPAL) Using New Micro‐Structured Charcoal Adsorbents Is Significantly More Effective in Removing Toxins and Improving Related Complications of Aoclf Than MARS‐a Multicenter Trial,” Hepatology 68, no. S1 (2018): 180A.

[21] T. Dethloff , F. Tofteng , H. J. Frederiksen , M. Hojskov , B. A. Hansen , and F. S. Larsen , “Effect of Prometheus Liver Assist System on Systemic Hemodynamics in Patients With Cirrhosis: A Randomized Controlled Study,” World Journal of Gastroenterology 14, no. 13 (2008): 2065–2071. 18395908 10.3748/wjg.14.2065 PMC2701529

[22] M. B. Iarustovskiĭ , M. V. Abramian , E. V. Komardina , et al., “Extracorporeal Methods of Hematological Correction in Patients With Acute Liver Insufficiency After Cardiac Surgery,” Anesteziologiia i Reanimatologiia 59, no. 5 (2014): 4–10. 25842933

[23] A. Kribben , G. Gerken , S. Haag , et al., “Effects of Fractionated Plasmaseparation and Adsorption on Survival in Patients With Acute‐On‐Chronicliver Failure,” Gastroenterology 142, no. 4 (2012): 782–789 e3, 10.1053/j.gastro.2011.12.056. 22248661

[24] D. Weil , E. Levesque , M. McPhail , et al., “Prognosis of Cirrhotic Patients Admitted to Intensive Care Unit: A Meta‐Analysis,” Annals of Intensive Care 7, no. 1 (2017): 33, 10.1186/s13613-017-0249-6. 28321803 PMC5359266

[25] R. Zhai , C. C. Sheu , L. Su , et al., “Serum Bilirubin Levels on ICU Admission Are Associated With ARDS Development and Mortality in Sepsis,” Thorax 64, no. 9 (2009): 784–790. 19482841 10.1136/thx.2009.113464 PMC2735615

[26] B. R. V. Kumar and S. Kumar Sarin , “Acute‐On‐Chronic Liver Failure: Terminology, Mechanisms and Management,” Clinical and Molecular Hepatology 29, no. 3 (2023): 670–689. 36938601 10.3350/cmh.2022.0103 PMC10366797

[27] A. Sekandarzad , E. Graf , E. P. Prager , et al., “Cytokine Adsorption in Patients With Acute‐On‐Chronic Liver Failure (CYTOHEP)—A Single Center, Open‐Label, Three‐Arm, Randomized, Controlled Intervention Pilot Trial,” Artificial Organs 48, no. 10 (2024): 1150–1161. 38770971 10.1111/aor.14774

