# Supplementary figures and images for: Comparison of Open Albumin Dialysis (OPAL) With Prometheus Fractionated Plasma Separation and Adsorption (FPSA) and Standard Medical Treatment for Acute‐On‐Chronic Liver Failure
Source: Artif Organs. 2025 Feb 25;49(6):997–1011. doi: 10.1111/aor.14977 (PMC12120814; doi:10.1111/aor.14977)

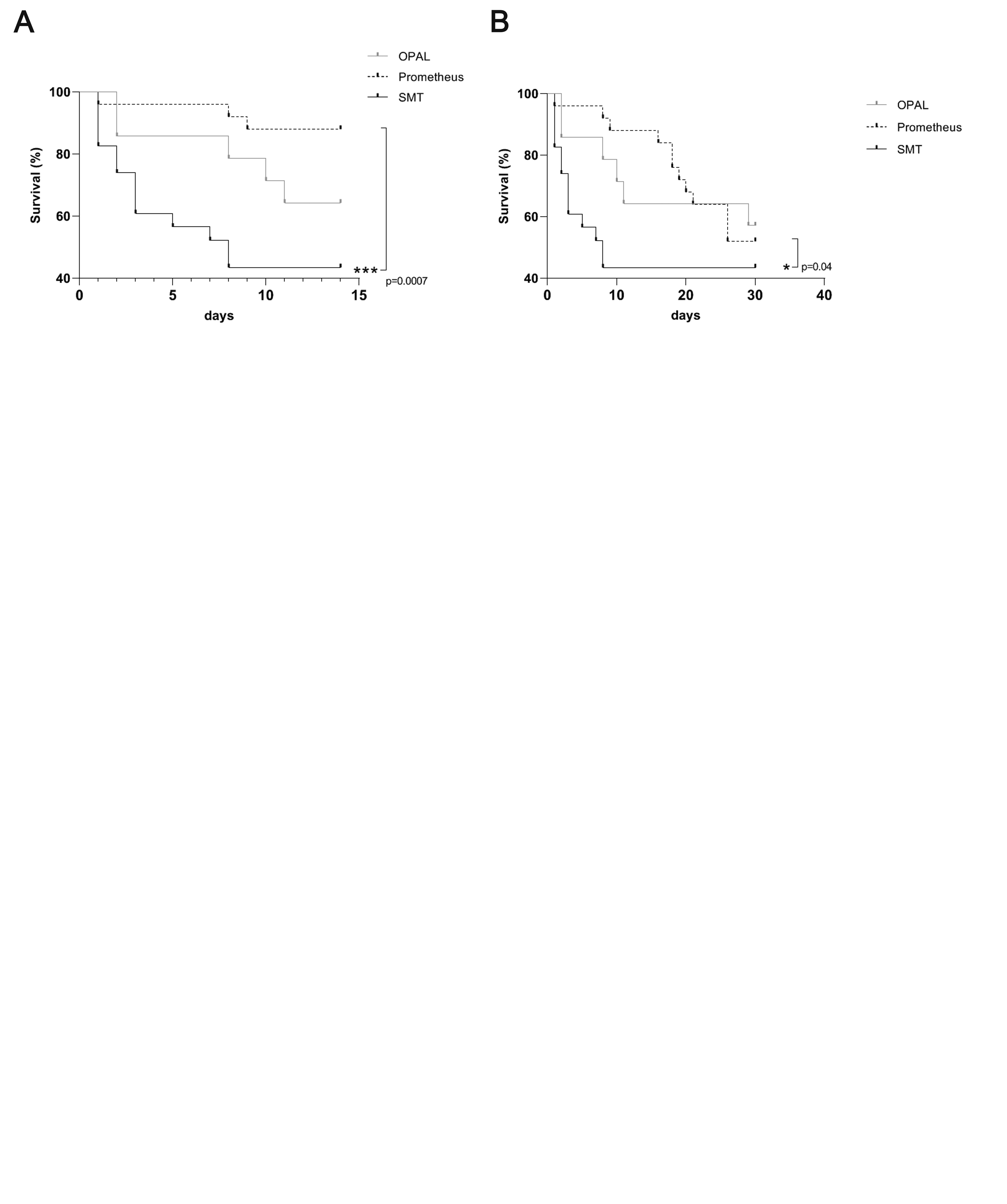

Supplement: Supplementary file 1 — Figure S1. Effect of treatment with various liver support procedures on overall mortality, considering only ACLF patients with acute kidney injury. (A) Short‐term 14‐day mortality rates comparing OPAL therapy with Prometheus therapy and with SMT plus hemodialysis. (B) Thirty‐day mortality rates comparing OPAL therapy with Prometheus therapy and with SMT plus hemodialysis. ACLF, acute‐on‐chronic liver failure; OPAL, open albumin dialysis; SMT, standard medical treatment. [file AOR-49-997-s001.tif]

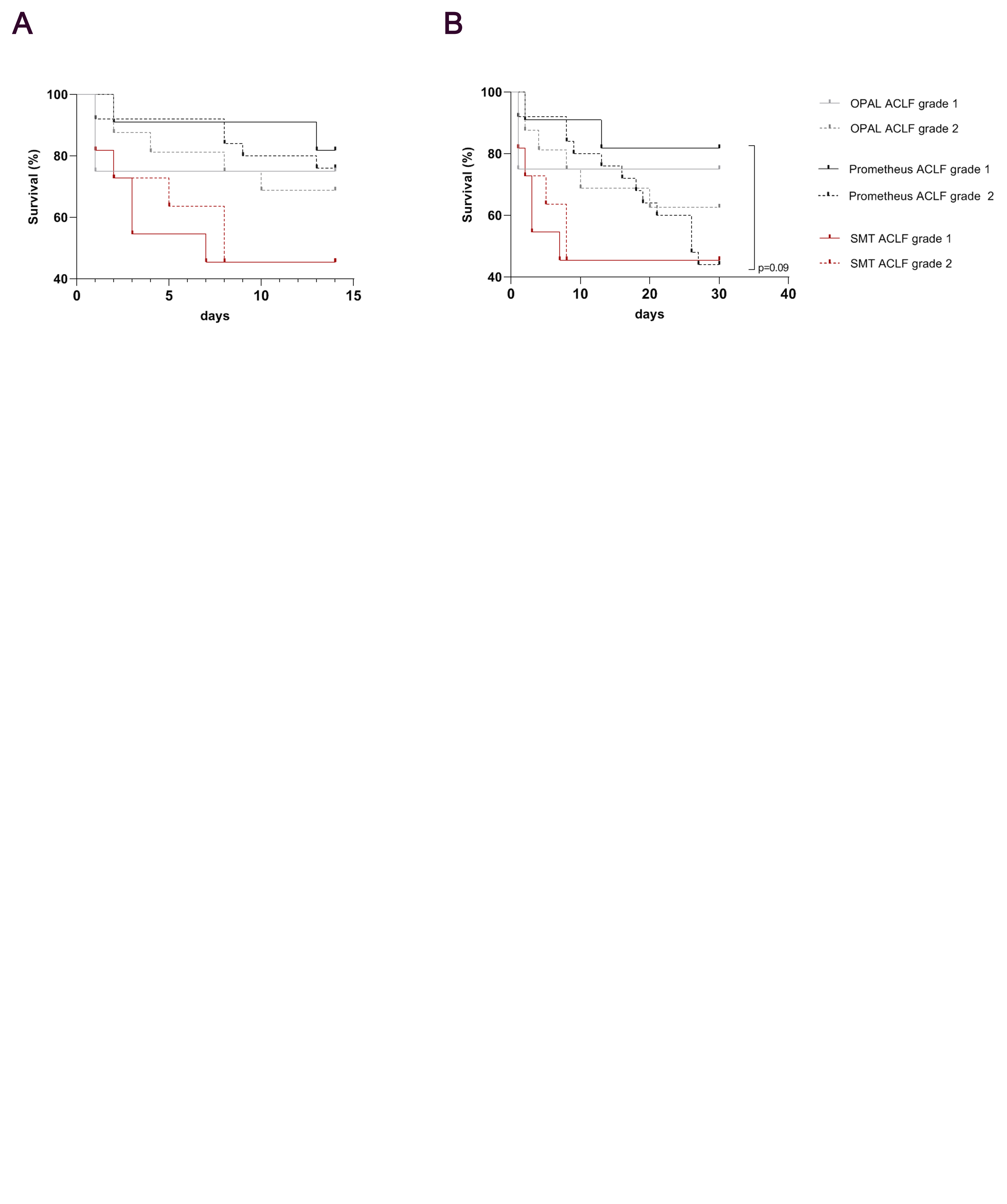

Supplement: Supplementary file 2 — Figure S2. Effect of treatment with various liver support procedures on overall mortality in dependence on ACLF grade. (A) Short‐term 14‐day mortality rates comparing OPAL therapy with Prometheus therapy and with SMT plus hemodialysis. (B) Thirty‐day mortality rates comparing OPAL therapy with Prometheus therapy and with SMT plus hemodialysis. ACLF, acute‐on‐chronic liver failure; OPAL, open albumin dialysis; SMT, standard medical treatment. [file AOR-49-997-s003.tif]
