# Supplementary material for: Comparison of Open Albumin Dialysis (OPAL) With Prometheus Fractionated Plasma Separation and Adsorption (FPSA) and Standard Medical Treatment for Acute‐On‐Chronic Liver Failure
Source: Artif Organs. 2025 Feb 25;49(6):997–1011. doi: 10.1111/aor.14977 (PMC12120814; doi:10.1111/aor.14977)
Supplement: Supplementary file 3 — Table S1. Comparison of baseline characteristics of 41 ACLF patients treated with Prometheus and 24 ACLF patients treated with SMT plus hemodialysis. [file AOR-49-997-s002.docx]

1. **Supplementary Material**

**Supplementary Table 1.** Comparison of baseline characteristics of 41 ACLF patients treated with Prometheus and 24 ACLF patients treated with SMT plus hemodialysis.

ACLF, acute-on-chronic liver failure; CI, confidence interval; ICU, intensive care unit; RR, relative risk; SMT, standard medical treatment

| **Variable** | **Prometheus  n = 41** | **SMT n = 24** | **RR (CI)** | **p value** |
| --- | --- | --- | --- | --- |
| Women, n (%) | 11 (27) | 11 (46) | 0.59 (0.3-1.2) | 0.12 |
| Multiorgan failure, n (%) | 30 (73) | 24 (100) | 1.73 (0.6-0.9) | **0.005** |
| Hepatorenal syndrome, n (%) | 26 (63) | 24 (100) | 0.63 (0.5-0.8) | **0.0007** |
| ACLF grade I, n (%) | 11 (27) | 12 (50) | 0.54 (0.3-1.0) | 0.06 |
| ACLF grade II, n (%) | 26 (63) | 11 (46) | 1.38 (0.9 -2.4) | 0.17 |
| ACLF grade III, n (%) | 4 (10) | 1 (4) | 2.34 (0.4-15.2) | 0.41 |
| ACLF due to infection, n (%) | 21 (51) | 16 (67) | 0.77 (0.5-1.2) | 0.23 |
| ACLF due to bleeding, n (%) | 4 (10) | 3 (13) | 0.78 (1.2-3.0) | 0.73 |
| ACLF due to alcohol consumption, n (%) | 12 (29) | 1 (4) | 7.02 (1.3-41.2) | **0.02** |
| ACLF due to other reasons, n (%) | 4 (10) | 4 (17) | 0.59 (0.2-2.0) | 0.41 |
| Hepatic encephalopathy, n (%) | 17 (42) | 12 (50) | 0.83 (0.5-1.5) | 0.50 |
| Dialysis before therapy initiation, n (%) | 11 (27) | 6 (25) | 1.07 (0.5-2.6) | 0.87 |
| Admittance to the ICU before therapy initiation, n (%) | 23 (56) | 12 (50) | 1.12 (0.7-1.9) | 0.63 |
| Another liver suport device before therapy initiation, n (%) | 1 (2) | 0 (0) | infinity  (0.2-infinity) | 0.44 |

**Supplementary Figure 1.** Effect of treatment with various liver support procedures on overall mortality, considering only ACLF patients with acute kidney injury. (A) Short-term 14-day mortality rates comparing OPAL therapy with Prometheus therapy and with SMT plus hemodialysis. (B) 30-day mortality rates comparing OPAL therapy with Prometheus therapy and with SMT plus hemodialysis.

ACLF, acute-on-chronic liver failure; OPAL, open albumin dialysis; SMT, standard medical treatment

**Supplementary Figure 2.** Effect of treatment with various liver support procedures on overall mortality in dependence on ACLF grade. (A) Short-term 14-day mortality rates comparing OPAL therapy with Prometheus therapy and with SMT plus hemodialysis. (B) 30-day mortality rates comparing OPAL therapy with Prometheus therapy and with SMT plus hemodialysis.

ACLF, acute-on-chronic liver failure; OPAL, open albumin dialysis; SMT, standard medical treatmen
